# Management of Malignant Gastric Outlet Obstruction: A Comprehensive Review on the Old, the Classic and the Innovative Approaches

**DOI:** 10.3390/medicina60040638

**Published:** 2024-04-16

**Authors:** Alessandro Fugazza, Marta Andreozzi, Hamid Asadzadeh Aghdaei, Agustin Insausti, Marco Spadaccini, Matteo Colombo, Silvia Carrara, Maria Terrin, Alessandro De Marco, Gianluca Franchellucci, Kareem Khalaf, Pardis Ketabi Moghadam, Chiara Ferrari, Andrea Anderloni, Giovanni Capretti, Gennaro Nappo, Alessandro Zerbi, Alessandro Repici

**Affiliations:** 1 Division of Gastroenterology and Digestive Endoscopy, Humanitas Research Hospital-IRCCS, Via Manzoni 56, Rozzano, 20089 Milan, Italy; alessandro.fugazza@humanitas.it (A.F.); marta.andreozzi@humanitas.it (M.A.); matteo.colombo@humanitas.it (M.C.); silvia.carrara@humanitas.it (S.C.); maria.terrin@humanitas.it (M.T.); alessandro.demarco@humanitas.it (A.D.M.); gianluca.franchellucci@humanitas.it (G.F.); alessandro.repici@hunimed.eu (A.R.); 2 Disorders Research Center, Research Institute for Gastroenterology and Liver Diseases, Shahid Beheshti University of Medical Sciences, Tehran P.O. Box 19875-17411, Iran; hamid.assadzadeh@gmail.com; 3 Department of Gastroenterology and Digestive Endoscopy, Medical Association Hospital, IGEA Institute, Patricios 347, Bahia Blanca B8000, Argentina; doctorinsausti@gmail.com; 4 Department of Biomedical Sciences, Humanitas University, 20090 Milan, Italy; giovanni.capretti@hunimed.eu (G.C.); gennaro.nappo@hunimed.eu (G.N.); alessandro.zerbi@hunimed.eu (A.Z.); 5 Division of Gastroenterology, St. Michael’s Hospital, University of Toronto, Toronto, ON M5B 1T8, Canada; kareem.khalaf@mail.utoronto.ca; 6 Research Institute for Gastroenterology and Liver Diseases, Shahid Beheshti University of Medical Sciences, Tehran P.O. Box 19875-17411, Iran; ketabimoghadam.p@gmail.com; 7 Division of Anaesthesiology, Humanitas Research Hospital-IRCCS, Via Manzoni 56, Rozzano, 20089 Milan, Italy; chiara.ferrari@humanitas.it; 8 Gastroenterology and Endoscopy Unit, Fondazione IRCCS Policlinico San Matteo, Viale Camillo Golgi 19, 27100 Pavia, Italy; a.anderloni@smatteo.pv.it; 9 Pancreatic Unit, Humanitas Research Hospital-IRCCS, Via Manzoni 56, Rozzano, 20089 Milan, Italy

**Keywords:** gastric outlet obstruction, EUS-guided gastrojejunostomy, duodenal stricture, gastroduodenal stenting, self-expanding metal stents

## Abstract

Gastrojejunostomy is the principal method of palliation for unresectable malignant gastric outlet obstructions (GOO). Gastrojejunostomy was traditionally performed as a surgical procedure with an open approach butrecently, notable progress in the development of minimally invasive procedures such as laparoscopic gastrojejunostomies have emerged. Additionally, advancements in endoscopic techniques, including endoscopic stenting (ES) and endoscopic ultrasound-guided gastroenterostomy (EUS-GE), are becoming more prominent. ES involves the placement of self-expandable metal stents (SEMS) to restore luminal patency. ES is commonly the first choice for patients deemed unfit for surgery or at high surgical risk. However, although ES leads to rapid improvement of symptoms, it carries limitations like higher stent dysfunction rates and the need for frequent re-interventions. Recently, EUS-GE has emerged as a potential alternative, combining the minimally invasive nature of the endoscopic approach with the long-lasting effects of a gastrojejunostomy. Having reviewed the advantages and disadvantages of these different techniques, this article aims to provide a comprehensive review regarding the management of unresectable malignant GOO.

## 1. Introduction

Malignant alimentary tract obstructions may occur in different parts of the digestive system, such as the distal esophagus, gastric cardia, distal stomach, small intestine, and colon. Malignant gastric outlet obstruction (GOO) specifically refers to an obstruction in the distal stomach, pylorus, or duodenum [1,2,3]. This condition may be accompanied with severe symptoms including postprandial vomiting, epigastric abdominal pain, bloating, discomfort, early satiety, and weight loss. If left untreated, it can lead to life-threatening emergencies [4,5]. The management of GOO ideally requires a multidisciplinary approach involving oncologists, surgeons, and endoscopists. It is crucial to recognize that symptoms of GOO can arise from both mechanical blockages and functional diseases. GOO is typically categorized into three main types based on its nature: benign mechanical obstructions, malignant mechanical obstructions, and functional obstructions/motility disorders. Distinguishing between these clinical entities can be challenging as they often present with similar features. In some cases, symptoms may only manifest in the later stages of the disease when the stomach has expanded significantly to accommodate larger volumes. Over time, there has been a shift in the primary cause of GOO from peptic ulcer disease, which was more prevalent in the past, to the current predominance of malignant causes. The development of GOO in case of malignant disease generally portends to an unfavorable prognosis [5,6], increased morbidity and diminished quality of life [6,7]. Furthermore, GOO can significantly impact the tolerability and effectiveness of oncologic treatments. Malignant GOO at the level of the pylorus or the duodenum are generally caused by malignancies involving the stomach, duodenum, pancreas, ampullary region or the biliary tree. GOO is caused by an underlying malignancy in up to 85% of patients, most commonly gastric or pancreatic cancer. Other uncommon causes of malignant GOO are gastric lymphomas, cystic neoplasms of the pancreas, gallbladder and bile duct adenocarcinomas, carcinoid tumors, retroperitoneal lymphadenopathies, retroperitoneal sarcomas, leiomyosarcoma, and gastrointestinal stromal tumors [4]

Diagnostic workup for GOO may include plain abdominal radiography, contrast upper gastrointestinal studies with Gastrografin or Barium and computed tomography (CT) with intravenous and oral contrast. Plain radiographs and upper GI studies can demonstrate the presence of gastric dilatation and identify the site of obstruction; an enlarged stomach with a narrowing of the pylorus or the first duodenal portion helps differentiate GOO from gastroparesis. Upper endoscopy is fundamental to directly identify the site of obstruction and provide tissue sampling when the obstruction is intraluminal. In the case of peri-gastric or peri-pancreatic malignancy, EUS-guided biopsy is used for establishing a preoperative diagnosis and to guide future management.

The management of GOO depends on the underlying etiology. Focusing on malignant GOO, surgical bypasses, endoscopic stenting (ES) with self-expandable metal stents (SEMS) and, more recently, endoscopic ultrasound-guided gastroenterostomy (EUS-GE), are the main therapeutic strategies. Surgical gastrojejunostomy has been the standard of care for the management of GOO for many years [8,9,10].

In the past, open surgical gastrojejunostomy was the primary approach for therapy. However, contemporary practices have shifted towards laparoscopic techniques, which offer significantly lower complication rates and shorter hospital stays [8]. The inherent advantage of a gastrojejunostomy lies in its permanence and fewer restrictions on post-interventional food texture and consistency [9]. Nevertheless, the patient’s fitness for such surgery is crucial, as frail individuals may experience delays in wound healing and restoration of bowel function. Historically, surgical gastrojejunostomy was considered the preferred therapy for patients with GOO who had a life expectancy exceeding 2 months and a good functional status [10]. However, a considerable number of patients with malignant GOO have advanced diseases and a limited life expectancy, making them unsuitable candidates for surgery [11]. Consequently, less invasive approaches have emerged as alternatives to surgical bypasses, aiming to provide rapid and effective symptom relief, enable comfortable oral feeding resumption, and achieve these goals with maximum safety, minimal hospitalization time, and reduced costs. Since the late 1990s, the placement of metal ES has proven to be more effective in early reintroduction of oral intake, reducing hospital stays, and minimizing major adverse events (AEs) compared to traditional surgery [12,13,14].

ES is typically reserved as a palliative measure, in cases such as unresectable lesions. ES is a safe option only when there is no evidence of a downstream disease. Issues with stent obstruction due to tumor ingrowth or food impaction can impose significant problems, particularly in patients whose treatment had significantly extended survival [15,16,17]. Following the rapid spread of EUS-guided drainage with lumen apposing metal stent (LAMS), EUS-GE was developed, with the first experimentations on animal models [18,19,20]. EUS-GE is a minimally invasive approach for patients with malignant GOO and to date seems to have a higher clinical success rate with a lower need for reinterventions when compared with ES. EUS-GE provides a hybrid solution of combining laparoscopic gastrojejunostomy and an endoscopic procedure; the added benefit stems from the less invasive nature of a surgical approach, making EUS-GE a more favorable option [9,21]. The purpose of this mini review is to provide an update on the latest available evidence in the literature regarding the surgical and the endoscopic techniques for the management of patients with malignant GOO.

## 2. Surgical Gastrojejunostomy Techniques

**Figure 1:**
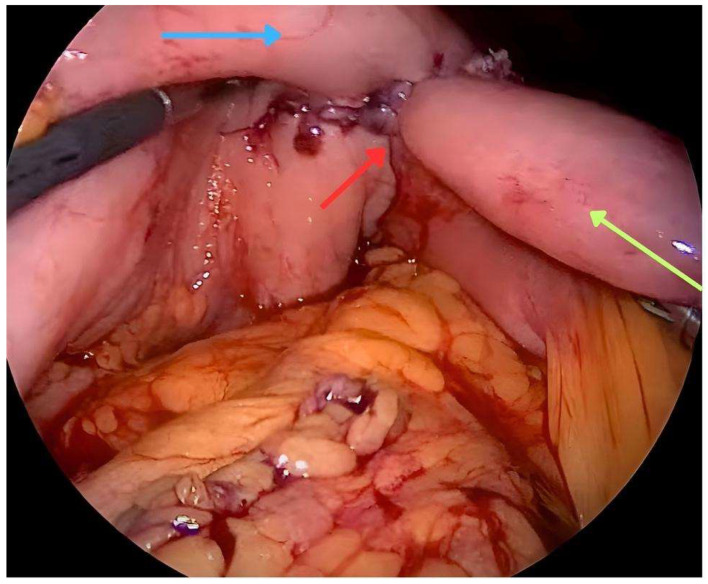
Laparoscopic view of gastrojejunostomy technique (LGJ). Anterior wall of the stomach (blue arrow). Anastomosis (red arrow). Loop of jejunum (green arrow). From the personal archive of the corresponding author.

Surgical gastrojejunostomy is a traditional procedure of gastrointestinal surgery, used to restore the continuity of the digestive tract in patients with GOO. This process can be carried out through open gastrojejunostomy (OGJ) or laparoscopic gastrojejunostomy (LGJ) techniques, each with its own outcomes and challenges. The OGJ technique has been the standard for decades. This approach has the advantage of providing a direct view of the surgical area and enabling precision in tissue connection. However, in a comparative study, the overall incidence of post-operative complications, classified as grade II or more according to Clavien-Dindo [22], was significantly higher in the OGJ group (30.4%) compared to the LGJ group (3.3%) (*p* = 0.015). LGJ has a lower incidence of surgical site infections and faster postoperative recovery compared to OGJ. Furthermore, the OGJ technique had worse results in terms of mean time until the resumption of oral feeding compared to LGJ (4 vs. 2 days; *p* < 0.001) [23] (Figure 1). However, there is not strong evidence regarding the two approaches. In a systematic review comparing surgical techniques (OGJ, LGJ) with ES for the management of patients with malignant GOO (20), ES was associated with a higher probability of tolerating oral food intake (OR 2.6) (*p* = 0.02), shorter time to reintroduce oral food intake (6.9 days, *p* < 0.001) and shorter post-procedure hospital stay (11.8 days, *p* < 0.001) [16]. On the other hand, no significant differences were observed in terms of 30 day mortality or survival.

## 3. Endoscopic Enteral Stenting

**Figure 2:**
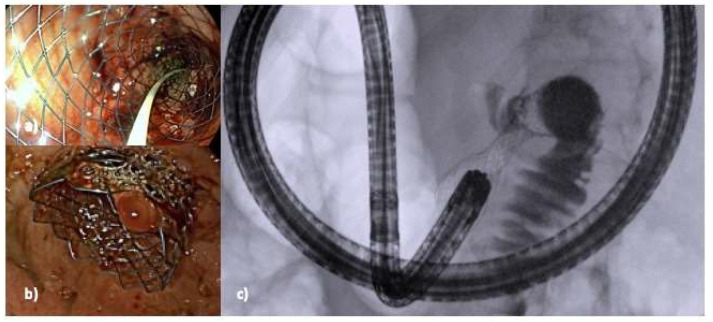
Placement of duodenal stent for duodenal stenosis. (**a**) Endoscopic image of the lumen of stent during release, (**b**) final endoscopic appearance of the proximal flange of stent, (**c**) final fluoroscopic appearance of the medium flowing freely from the gastric cavity to the distal duodenum through the stent. From the personal archive of the corresponding author.

**Figure 3:**
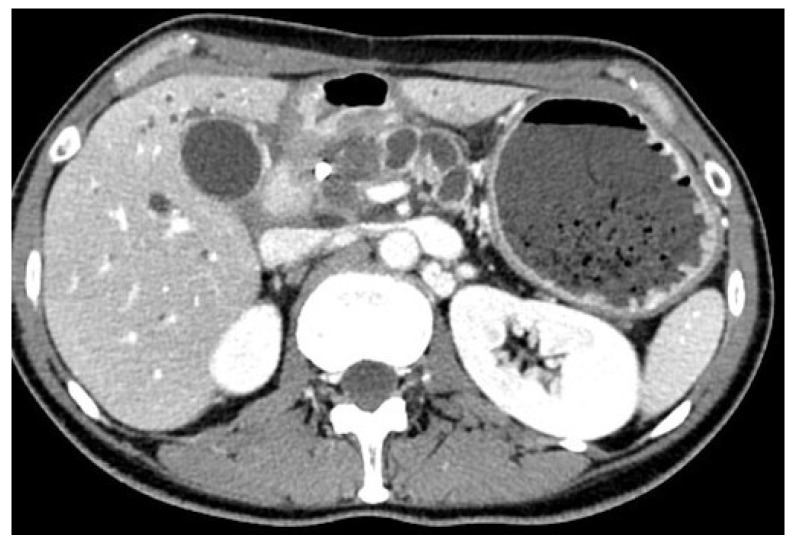
Computed tomography scan: a case of concomitant biliary and duodenal obstruction. From the personal archive of the corresponding author.

Endoscopic stent placement has emerged as a minimally invasive alternative to surgical intervention for the management of different gastrointestinal conditions. This technique involves the placement of either a plastic or metallic stent through an endoscope at the site of the narrowed stenosis to reopen the blocked passage [24,25]. The procedure can be conducted with the patient under conscious or deep sedation. It necessitates precise placement within the obstructed segment of the gastrointestinal tract, typically using both endoscopic visualization and fluoroscopic guidance for accuracy [13,14]. Currently, SEMS are the most commonly utilized type of gastroduodenal stents. They are made of thin, flexible metal wires, usually composed of nitinol, and they are designed to be deployed in a collapsed form and then gradually expand to their desired diameter once released [26,27]. Fully covered SEMS have an additional covering made of silicone or polytetrafluoroethylene (PTFE) which prevents tissue ingrowth, prolonging stent patency and reducing the rate of reinterventions [28]. Most of the gastroduodenal stents require a therapeutic working channel endoscope so that the stent may be passed through the scope. This method allows the endoscopist to visualize the area directly and adjust the stent placement as needed. Fluoroscopy is useful in combination with endoscopy to guide the stent placement in the correct position. During the procedure, a wire is inserted through the stricture using a catheter under endoscopic and fluoroscopic assistance. The use of a catheter over the wire allows for the injection of a contrast to better define the anatomy of the stenosis and optimize the size of the stent [29,30]. Depending on the endoscopist’s preference, a soft-tip wire may be used initially to pass the stricture, and then exchanged with a stiffer one. Then, a SEMS is passed over the wire through the scope and released across the stenosis under endoscopic and fluoroscopic control (Figure 2). ES with SEMS showed a high rate of technical success and a good rate of clinical success for the relief of obstructive symptoms and an improvement in food oral intake in patients affected by malignant GOO. The Gastric Outlet Obstruction Scoring System (GOOSS) is often used to quantify the clinical improvement after the treatment. However, there is heterogeneity among studies in the definition of this outcome. According to some reviews, the technical success and clinical success rate of ES is about 97% and 89%, respectively. The mean GOOSS improved from 0.4 to 2.4 after treatment, and symptoms were resolved within an average of 4 days [31,32]. Several studies have investigated factors that may predict clinical failure or stent dysfunction in order to improve patient outcomes. Retrospective studies found that peritoneal carcinomatosis is only a predictive factor of failure if accompanied by ascites, and that patients with peritoneal carcinomatosis without ascites did not have a lower clinical success rate compared to those without peritoneal disease. The location and number of strictures in the gastro-duodenal area are also associated with worse outcomes in retrospective studies [33,34,35]. The rate of adverse events associated with the placement of SEMS can vary from 0 to 30%, depending on how they are defined in the different studies. They can range from minor adverse events, such as nausea and vomiting, to major ones, including bleeding, perforation, pancreatitis, stent migration, and cholangitis. Delayed complications are often related to stent dysfunction caused by migration or obstruction from food impaction or tumor ingrowth [36]. In a large study including 1281 patients with duodenal SEMS placement, stent obstruction occurred in 12.6% of cases, migration in 4.3%, bleeding in 4.1% (with major bleeding in 0.8%), and perforation in 1.2%. Another study of 220 patients reported a SEMS-related adverse events rate of 2%, with three cases of fatal perforation and a re-intervention rate of 13% after 4 months. Stent occlusion can lead to the recurrence of symptoms and may require endoscopic re-intervention, which can negatively impact patients’ quality of life and increase healthcare costs [37,38]. To reduce the risk of stent occlusion, covered SEMS have been investigated and a meta-analysis of nine studies involving 849 patients showed that covered SEMS have a lower obstruction rate, but a higher risk of migration compared to uncovered SEMS [39]. The study [39] found no significant differences between the covered and uncovered SEMS groups in terms of technical success rate, clinical success rate, post-stenting dysphagia score, stent patency, overall complications, and re-intervention rate [39]. A more recent systematic review also reported similar results, showing comparable technical and clinical success rates between covered and uncovered gastroduodenal SEMS. However, the authors highlighted a trend towards a lower dysfunction rate in the covered SEMS group, but a higher risk of migration and overall adverse events rate in the same group. Some technical modifications and precautions have been proposed to prevent stent migration, but covered SEMS still carry a higher risk of cholangitis and pancreatitis. Uncovered SEMS are still considered to be the first option in this situation [40]. In advanced cases of gastro-duodenal or pancreato-biliary cancers, biliary obstruction frequently occurs, making standard endoscopic drainage via endoscopic retrograde cholangiopancreatography (ERCP) difficult. In patients facing simultaneous biliary and duodenal obstructions, and if the papilla remains reachable, considering ERCP following the placement of a duodenal SEMS may be a viable option. Nonetheless, conducting ERCP through a placed duodenal stent presents technical challenges. The advent of interventional EUS has revolutionized the management of patients with concurrent biliary obstruction and GOO. EUS-guided biliary drainage (EUS-BD) has proven to be a safe and effective method, even when performed alongside or through an existing duodenal stent, and is now regarded as the preferred strategy for biliary drainage in this group of patients (Figure 3) [41,42,43,44].

## 4. EUS-Guided Gastroenteric-Anastomosis

In recent years, EUS-GE utilizing LAMS has become a prominent minimally invasive approach for treating GOO. This technique boasts numerous benefits, such as the possibility of conducting it under conscious sedation, the use of real-time imaging for guidance, and the accurate placement of stents. It effectively alleviates symptoms, enables the resumption of oral intake, and enhances the quality of life for patients suffering from GOO. Varying in terms of length and diameter, all LAMS present a unique feature, which is their bi-flanged shape which ensures the proper approximation of two lumens, thereby minimizing the potential risk of leaks and perforation [45]. Additionally, LAMS are designed to distribute pressure evenly along the new-born fistulous tract, providing stability and minimizing the risk of stent migration. Moreover, these stents are completely covered, which prevents leakage through the anastomosis and reduces the chances of tissue ingrowth. The introduction of the electrocautery-enhanced LAMS (EC-LAMS) allowed a single-step procedure with the direct passage of the catheter into the target structure without prior access and tract dilation, thus avoiding device exchange [46,47]. This approach potentially reduces the risk of complications and procedure failure by minimizing the number of steps required for device exchanges. This is particularly important in certain steps like wire advancement and tract dilation, which can inadvertently cause the movement of the target jejunal loop away from the stomach during the procedure. The Hot-Axios™ (Boston Scientific Corporation, Natick, MA, USA) was the first EC-LAMS released into the market, leading to its widespread adoption. Recently, an EC-LAMS version by Taewoong Medical (Hot-Spaxus; Taewoong Medical Co., Gimpo, Republic of Korea) has been released into the market [48]. Stent deployment occurs through the gradual release of each flange while being visualized endoscopically and endosonographically. Although fluoroscopy is not obligatory, it is highly recommended during the procedure as it provides an additional visualization for safety purposes. EUS-GE may be performed with two major techniques: the direct unassisted and the device assisted EUS-GE.

## 5. Technical Aspects

### 5.1. Direct Unassisted EUS-GE

Direct unassisted EUS-GE is favorable in scenarios where there is complete luminal obstruction leading to the inability to traverse the stenosis with an endoscope or a guidewire. In this method, the bowel distal to the obstruction is punctured under EUS guidance and distended through a 19 gauge (0.91 mm, 3.58 inch) or 22 gauge (0.64 mm, 2,52 inch) needle with at least 200 cm^3^ of saline solution, mixed with contrast as well as a staining agent. The contrast allows for the visualization of the target bowel loop using fluoroscopy while the methylene blue enables the confirmation of the position. However, visualizing the intestinal loop through EUS may be challenging due to partial distention or distortion caused by intraluminal air. In cases where there is excessive peristalsis, anti-peristaltic drugs like glucagon or butyl-scopolamine may be considered [8,49,50]. Once the target jejunal limb is clearly identified using EUS, a safe needle puncture is performed using a 19 gauge (0.91 mm, 3.58 inch) or 22 gauge (0.64 mm, 2,52 inch) needle with consequent aspiration and injection. The large volume distension of the bowel provides a wider, and therefore, safer, landing zone for the direct, freehand insertion of the EC-LAMS catheter and subsequent deployment of the stent [51,52]. Mastery of the free-hand technique is strongly recommended prior to embarking on this procedure [52,53]. Once access is achieved, it is crucial to avoid the urge to insert a wire, as this action could cause the bowel wall to be displaced from the stomach wall. The potential hazards associated with this method include the minimal distension of the jejunal loop, which can lead to insufficient access for the EC-LAMS and the subsequent drift of the small bowel loop away from the stomach wall, along with the risk of incorrect stent placement.

### 5.2. Device-Assisted EUS-GE

#### 5.2.1. Ultraslim Endoscope-Assisted Technique

In this method, an ultra-slim endoscope is carefully guided through the stricture to directly visualize the distal small bowel and fluid administered directly through the endoscope to distend the target bowel loop. Subsequently, an echoendoscope is inserted and advanced into the stomach alongside the ultra-slim endoscope and, under EUS guide, the distended bowel loop is punctured and a guide wire of either 0.025 inches (0.64 mm) or 0.035 inches (0.89 mm) is introduced and coiled within the jejunum. If the wire can be observed endoscopically, it may be pulled through the ultra-slim endoscope with a forceps, providing added tension and traction during LAMS placement as a sort of an internal rendezvous [8,45,54]. However, this technique comes with some limitations. Firstly, this approach necessitates the setup of two separate endoscopic processors to enable the simultaneous operation of the ultra-slim and echoendoscope, which may not be readily available in all endoscopy units. Additionally, the insertion and introduction of the echoendoscope alongside the ultra-slim endoscope may be challenging, and often requires two operators to control each scope.

#### 5.2.2. Endoscopic Ultrasound Double Balloon-Assisted Technique

Initially, a standard endoscope is introduced alongside an overtube designed for a double-balloon enteroscope, navigating past the stenotic segment. The overtube aids in preventing the double-balloon enteric tube from looping in the stomach’s fornix, facilitating its passage through the pyloric-duodenal stenosis. A stiff 0.025 inches (0.64 mm) or 0.035 inches (0.89 mm) guidewire is advanced into the jejunum using an ERCP catheter through the working channel of the scope. Subsequently, the endoscope is removed while the over-tube and the guidewire are kept in place. Then, the tube in the jejunum is positioned where the stent will be placed, guided using fluoroscopy. A small amount of contrast medium and saline are injected into each balloon simultaneously to prevent their movement. The injection of saline is continued until each balloon changes from a spherical shape to a barrel shape. The over-tube is then gently removed and an echoendoscope is advanced into the stomach. The target jejunum is visualized and punctured between the two balloons using EUS after irrigating with tap water or saline mixed with contrast medium. The process of irrigation is continued until the target jejunum is sufficiently distended and can be seen clearly on EUS and fluoroscopy. Lastly, the EC-LAMS delivery system is used to deliver the stent between the gastric and the target jejunum [8].

#### 5.2.3. Nasobiliary Drain-Assisted Technique

**Figure 4:**
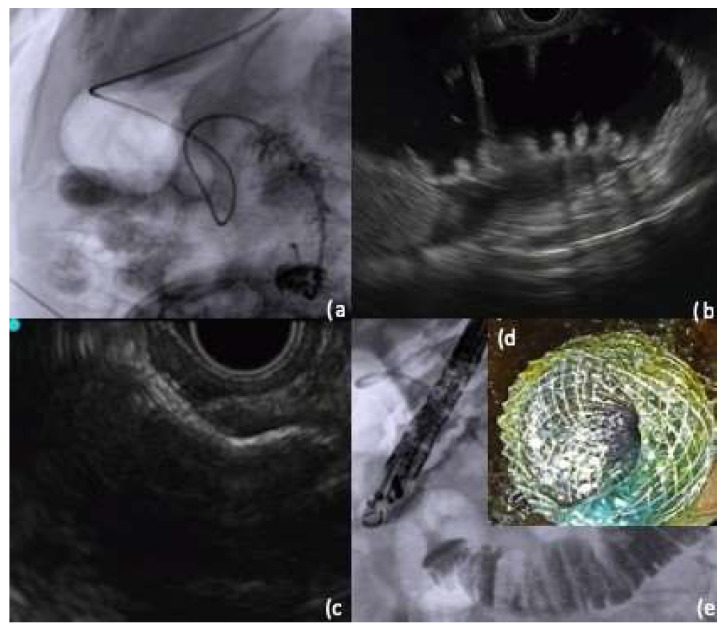
Gastroenteroanastomosis using lumen apposing metal stent (LAMS) with nasobiliary drain-assisted technique; (**a**) nasobiliary tube placement through the stenosis under fluoroscopic control; (**b**) endoscopic ultrasound (EUS) image of the target jejunal loop distended using fluid administration through the nasobiliary tube; (**c**) EUS image of first flange release of the LAMS; (**d**) final endoscopic image with methylene blue coming from the jejunum through the LAMS; (**e**) fluoroscopic image with contrast medium flowing freely from the gastric cavity to the jejunal loop through the LAMS. From the personal archive of the corresponding author.

Similar to the technique involving a balloon device, a guidewire is initially inserted and advanced across the stricture, coiling it in the small intestine beyond the obstruction. Once the guidewire is appropriately positioned, the endoscope is withdrawn from the patient’s mouth, and only under fluoroscopic guidance, the nasobiliary drain is advanced. Alternatively, a therapeutic endoscope or ERCP scope can be used to assist in placing the nasobiliary drain. The larger channel size of these endoscopes allow for the direct insertion of the drain through the endoscope channel and across the obstruction. The contrast medium can be injected through the nasobiliary drain to confirm its positioning in the desired loop of bowel. The nasobiliary drain can then be connected to an irrigation pump activated using a foot pedal, allowing for the continuous infusion of fluid during EUS-GE. The use of an irrigation pump provides the advantage of delivering a consistent flow of fluid distal to the obstruction, thus creating a more reliable target loop for subsequent EUS-guided access and LAMS positioning (Figure 4) [55].

#### 5.2.4. Endoscopic Ultrasound-Guided Double Balloon-Occluded Gastrojejunostomy Bypass

The endoscopic ultrasonography-guided double balloon-occluded gastrojejunostomy bypass (EPASS) has been introduced as an alternative technique for EUS-GE [8]. In this procedure, a unique double-balloon enteric tube (Tokyo Medical University type; Create Medic Co., Ltd., Yokohama, Japan) is utilized. This device, which is advanced through the stricture over a 0.64025 inch (0.64 mm) or 0.035 inch (0.89 mm) guidewire, is equipped with two balloons that are inflated using contrast medium. Fluid is also used to bring the target jejunal loop closer to the stomach wall and simultaneously distend it allowing for a larger diameter target and a stable position in the desired location. It is important to note that this balloon is not available outside of Japan thus limiting the applicability of this technique [56,57,58].

## 6. EUS-GE Outcomes and Comparison to Other Techniques

**Figure 5:**
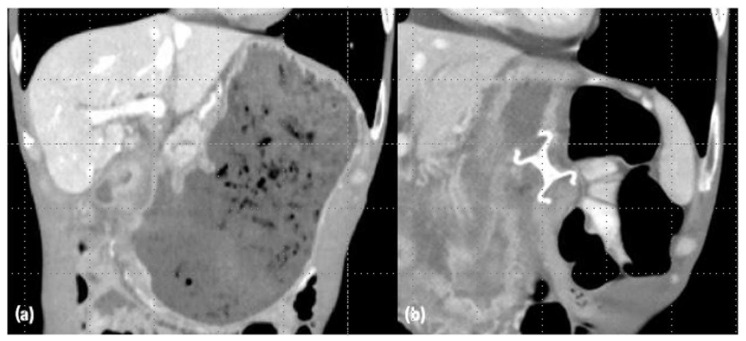
CT scan: (**a**) a case of significant gastrectasia due to tight neoplastic pyloric stricture; (**b**) same patient after positioning of EUS-GE with LAMS (visible in the image): the stomach is empty, and the stent is patent.

Studies evaluating the outcomes of EUS-GE for GOO treatment have consistently reported high rates of technical success (86.7–100%), defined as achieving proper positioning and deployment of the stent regardless of the specific technique utilized [56,57] (Figure 5). Similarly, clinical success rates, which measure the patient’s ability to tolerate oral intake or improvement in the GOOSS by at least 1 point, ranged between 80% and 100% [57,58,59,60,61,62]. Adverse events have also been documented in these studies, with reported rates varying from 0 to 26%. Common reported AEs include the misdeployment of the stent, peritonitis, bleeding, hemo- and pneumoperitoneum, abdominal pain, and leakage [60]. McCarty et al. recently conducted a systematic review and meta-analysis to assess the effectiveness and safety of EUS-GE in the management of benign and malignant GOOs. Their analysis included one prospective study and four retrospective studies, with a total of 199 patients. The average procedure time for both assisted and unassisted EUS-GE techniques was 43.5 ± 20 min, and most patients (*n* = 198; 99.5%) received a 15 × 10 mm LAMS. The pooled rates of technical success and clinical success were 92.9% and 90.1%, respectively. The overall adverse events rate was 10.6%, with serious events occurring in 5.6% of cases [58]. Two studies investigated predictors of technical success. Tyberg et al. found that previous intervention, altered anatomy, and the use of LAMS with or without cautery did not significantly impact technical outcomes. However, the presence of moderate or severe ascites was associated with lower technical success (42.9%) compared to patients with mild or no ascites. A multivariate analysis revealed that the distance between the lumen was the only predictor of technical success, with an optimal distance identified at 19 mm [63]. Regarding the different EUS-GE techniques, a comparative study has shown that the direct unassisted method achieves comparable technical and clinical success rates with a similar safety profile to balloon-assisted EUS-GE with shorter procedural time [53]. With the increasing diffusion of EUS-GE in recent years, several studies comparing its effectiveness with other techniques such as surgical GJ and ES have been conducted. A retrospective study involving 54 patients from four academic centers in three countries found comparable efficacy between LGJ and EUS-GE, but EUS-GE was associated with significantly fewer adverse events [64,65].

Two recent studies have compared the outcomes of EUS-GE with surgical bypass and luminal stenting for gastric outlet obstruction (GOO). In the comparison with surgical bypass, although EUS-GE had a lower technical success rate (86.7% vs. 100%), there was no significant difference in clinical success rates (87% vs. 90%) [45,66]. The recurrence of GOO and AEs rates were comparable between the two groups [67]. Additionally, EUS-GE was reported to be significantly less costly than surgery. EUS-GE also seem to have some advantages when compared to the traditional ES. A multicenter retrospective study on 82 patients comparing ES to EUS-GE found no significant differences in technical success, clinical success, or AEs, but EUS-GE had significantly lower rates of symptom recurrence and need for re-intervention [65]. Recently, important results from an international, multicenter, randomized, controlled trial on 185 patients with GOO treated with EUS-GE vs. ES have been published. In patients with malignant GOO, EUS-GE reduced the frequency of intervention, improved stent patency, and resulted in better patient-reported eating habits compared with ES. No statistically significant differences in technical success, clinical success, AEs rate or quality-of-life scores at 1 month and death within 30 days were reported. This work strongly suggests that EUS-GE should be used preferentially over ES when expertise and dedicated devices are available [68]. Based on the currently available data, EUS-GE is considered effective and safe for malignant GOO, posing as an alternative option to surgery or luminal stenting in suitable cases when performed by experienced endoscopists. For patients with advanced disease and short life expectancy, luminal stenting may be preferred (Table 1).

## 7. Conclusions

EUS-GE is becoming recognized as an effective and safe method for managing malignant GOO syndrome. Relative to ES, EUS-GE potentially provides a more durable option, melding the benefits of surgical bypass with the minimal invasiveness characteristic of endoscopic approaches. Nevertheless, for patients presenting with malignant GOO who have a limited life expectancy and poor performance status, the deployment of SEMS remains the recommended strategy, taking into account considerations of safety, effectiveness, and cost. The choice between uncovered and covered SEMS should be based on patient-related factors such as the location of the stenosis, tumor type and the involvement of the papilla or the common bile duct by the tumor. Surgical GJ undoubtedly guarantees durable results and low rates of long-term complications and reinterventions. Surgical GJ should be considered in fit patients with a life expectancy of >2 years. EUS-GE LGJ demonstrate similar technical and clinical success rates. However, the shorter time to oral intake, reduced hospital stays, and a lower incidence of adverse events suggest that EUS-GE should be preferred when expertise and devices are available. On the other hand, it is important to note that significant AEs can occur if the procedure is not performed by experts and proper patient selection and procedural expertise are essential to minimize complications and optimize outcomes.

## Figures and Tables

**Table 1:** Advantages and disadvantages of the different techniques for GOO management.

Technique	Advantages	Disadvantages
Surgical gastrojejunostomy [16,17,21,66]	▪ Effective ▪ Can last lifetime	▪ Invasive ▪ Prolonged hospitalization ▪ Not suitable for patients in poor clinical conditions
Endoscopic Stenting (ES) [9,16,21,30,31,68]	▪ Less invasive than surgery ▪ Quick relief of symptoms	▪ High obstruction/migration rates
Endoscopic ultrasound-guided gastroenterostomy (EUS-GE) [56,57,58,59,60,61,62,66,68]	▪ Less invasive than surgery ▪ Long lasting results	▪ Not suitable for all patients (ascites, carcinomatosis…) ▪ Need high expertise

## Data Availability

No new data were created or analyzed in this study. Data sharing is not applicable to this article.
